# Pediatric Diabetic Nephropathy: Novel Insights from microRNAs

**DOI:** 10.3390/jcm12041447

**Published:** 2023-02-11

**Authors:** Francesca Lanzaro, Annalisa Barlabà, Angelica De Nigris, Federica Di Domenico, Valentina Verde, Emanuele Miraglia del Giudice, Anna Di Sessa

**Affiliations:** Department of Woman, Child, and General and Specialized Surgery, University of Campania “Luigi Vanvitelli”, 80138 Naples, Italy

**Keywords:** diabetic, nephropathy, microRNA, treatment, biomarker, children

## Abstract

Diabetic nephropathy (DN) represents the most common microvascular complication in patients with diabetes. This progressive kidney disease has been recognized as the major cause of end-stage renal disease with higher morbidity and mortality. However, its tangled pathophysiology is still not fully known. Due to the serious health burden of DN, novel potential biomarkers have been proposed to improve early identification of the disease. In this complex landscape, several lines of evidence supported a critical role of microRNAs (miRNAs) in regulating posttranscriptional levels of protein-coding genes involved in DN pathophysiology. Indeed, intriguing data showed that deregulation of certain miRNAs (e.g., miRNAs 21, -25, -92, -210, -126, -216, and -377) were pathogenically linked to the onset and the progression of DN, suggesting not only a role as early biomarkers but also as potential therapeutic targets. To date, these regulatory biomolecules represent the most promising diagnostic and therapeutic options for DN in adult patients, while similar pediatric evidence is still limited. More, findings from these elegant studies, although promising, need to be deeper investigated in larger validation studies. In an attempt to provide a comprehensive pediatric overview in the field, we aimed to summarize the most recent evidence on the emerging role of miRNAs in pediatric DN pathophysiology.

## 1. Introduction

As the increasing prevalence of diabetes worldwide [1,2,3], its cardiometabolic consequences have received remarkable scientific attention since childhood [4,5,6]. In particular, diabetic nephropathy (DN) represents one of the most important long-term complications of diabetes [7,8], and is currently recognized as the major cause of end-stage renal disease (ESRD) with high morbidity and mortality rates [1,4,9].

**Figure 1:**
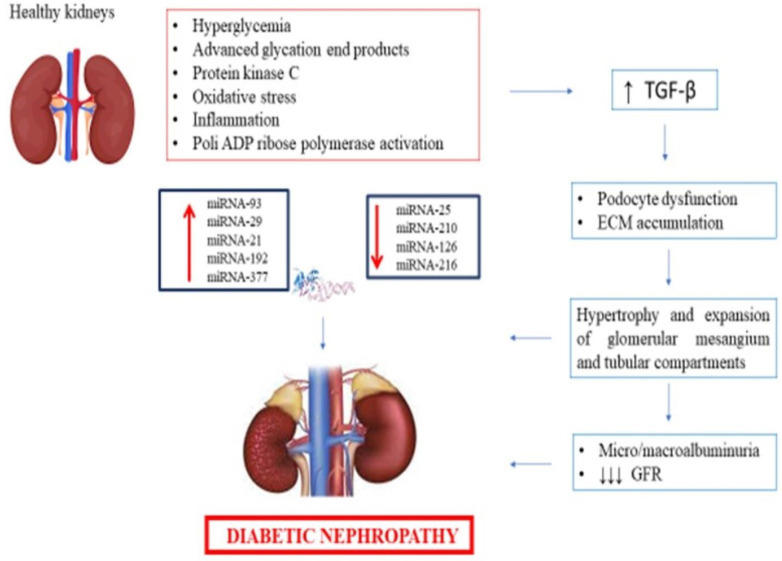
The role of miRNAs in the pathophysiology of diabetic nephropathy. Abbreviations: ECM: extracellular matrix; GFR: glomerular filtration rate; TGF-*β*: transforming growth factor-*β*.

Both persistent albuminuria and reduced glomerular filtration rate (eGFR) represent the main clinical features of the disease. In the early stages of DN, microalbuminuria (defined as the presence of 30–300 mg a day of albumin in urine) is detected in these patients, while albuminuria (>300 mg/day) appeared during the progression of the disease [10]. Various pathophysiological mechanisms have been supposed in DN development including hyperglycemia, inflammation, oxidative stress, advanced glycation end products, protein kinase C, and poly(ADP-ribose) polymerase activation [11,12,13] (Figure 1). Taken together, these factors are responsible for the morphological impairments occurring at renal site in DN, such as glomerular mesangium hypertrophy, podocytes dysfunction, and extracellular matrix proteins accumulation [13,14]. From a molecular perspective, different cellular and inflammatory signaling pathways such as transforming growth factor-*β* (TGF-*β*), Phosphoinositide 3-kinase-protein kinase B (PI3K-Akt), Mitogen-activated protein kinase (MAPK) family including P38, extracellular signal-regulated kinases (ERK), Nuclear factor kappa-light-chain-enhancer of activated B cells (NF-*κ*B), and c-Jun N-terminal kinases (JNKs) have been implied in DN pathogenesis [13,15].

Given the serious cardiometabolic burden of DN, research efforts are recently focusing on novel potential biomarkers to improve early identification of the disease [10,16,17,18].

Besides classical markers (e.g., urinary cystatin C) [10,19,20,21], novel promising options are emerging in this research area [14,20,22]. In this framework, a pathogenic role for microRNAs (miRNAs) as biomolecules regulating up to 30% of the protein-coding genes in the human genome has been also proposed [23,24,25,26]. To date, miRNAs represent the most attractive candidates as potential diagnostic, prognostic, and therapeutic DN tool [27,28,29].

Similar to other conditions (including non-Alcoholic fatty liver disease (NAFLD), etc.) [30,31], miRNAs up-regulation has been supposed to contribute to the development and progression of the disease [29,32]. Particularly, several different miRNAs, such as miRNA-21, -25, -29, -210, -216, -126, -377, and -92 have been pathogenically linked to DN [33,34,35].

To date, most evidence in this attractive research area is based on adult [36,37,38,39,40] and in vitro studies [27,28,41,42], while similar pediatric data are still limited [43,44,45,46,47]. Due to the urgent need for early identification of DN and of the fragmentariness of data in the field in childhood, we attempted to provide a comprehensive pediatric overview on the challenging role of miRNAs in DN.

## 2. Evidence on the Role of miRNAs -21, -126, -216, and -377 in DN Pathogenesis

Recent advances have reported the pivotal role of certain miRNAs in DN pathogenesis [43,45,46,48] (Table 1). 

These biomolecules—transported by macrovesicles, exosomes, and transmembrane proteins—are involved in the regulation of different pathways promoting fibrosis and inflammation especially in the heart and the kidney [12,20,24]. As the lack of influence of glomerular filtration on their urinary levels unlike serum creatinine [49], miRNAs can be used as innovative biomarker for DN even at the preclinical stage [25,27,28].

MiRNA-21, a well-known type 2 diabetes (T2D)-associated miRNA [50,51], is a significant microRNA particularly involved in cardiac [52] and renal fibrosis [53,54,55,56,57]. Upregulation of its levels has been detected in human renal diseases [53,54] by promoting TGF-β-mediated effects on endothelial-to-mesenchymal transition [55]. In this context, a large amount of data focused on dysregulation of miRNA-21 in diabetes and kidney injury [45,56,57,58,59], suggesting its potential role as accessible biomarker and novel target for diagnosis and treatment of T2D renal consequences [56].

Preclinical studies have attempted to explain which pathways are modified by miRNA in kidney disease [57,58,59]. Dey et al. [57] showed that miRNA-21 downregulated the Target of rapamycin complex 1 (TORC1)-pathway in a type 1 diabetes (T1D) mouse model with renal disease during hyperglycemia. Wang et al. [58] suggested that renal fibrosis in T2D mice was the effect of modulated metallopeptidase. In particular, miRNA-21 contributed to renal fibrosis downregulating matrix metallopeptidase9 (MMP9)/tissue inhibitor matrix metalloproteinase-1 (TIMP-1) [58]. Zhong et al. [59] demonstrated that renal miRNA-21 expression was upregulated in T2D mice models and was linked to the onset of microalbuminuria, renal fibrosis, and inflammation. Remarkably, transfer of miRNA-21 knockdown plasmids into the diabetic kidneys of db/db mice resulted in a significant decrease in renal fibrosis and inflammation [59]. Moreover, decapentaplegic homolog 7 (SMAD7), a protective factor in renal fibrosis and inflammation through TGF-ß and NF-κB pathways suppression, was inversely linked to miRNA-21. In db/db mice, it was found to be significantly decreased during diabetic renal damage and partially improved when miRNA-21 was suppressed [59].

On the other hand, two cross-sectional observational cohort study [45,51] investigated the role of different miRNAs, such as miRNA-21, miRNA-126, and miRNA-210, in a large cohort of children with T1D [45], and with T2D [51]. Compared to pre-clinical data, Osipova et al. [45] reported a significant upregulation of plasma and urinary miRNA-21 and miRNA-210 levels in a population of 68 children and adolescents aged 6–18 years with T1D compared to 79 age- and gender-matched healthy subjects without relevant cardiovascular risk factors [45]. Both plasma and urinary miRNA-21 and miRNA-210 levels were found to be increased in patients with T1D than in healthy subjects (*p* = 0.008 and *p* = 0.0001 for plasma values, respectively; *p* < 0.0001 and *p* = 0.002 for urinary values, respectively) [45]. In contrast with previous clinical evidence for T2D [60], no difference in plasma miR-126 levels were found [45], whereas urinary miRNA-126 concentrations were significantly decreased in these patients (*p* = 0.016) [45]. Moreover, a significant association between glycated hemoglobin (HbA1c) mean and urinary miRNA-126 concentrations in subjects with T1D was demonstrated (r = −0.286, *p* = 0.042) [45].

As potential indicator of persistent renal inflammation in patients with T1D, a positive correlation between C-reactive protein (CRP) and urinary miRNA-21 levels was also reported [45]. In line with previous data supporting early diabetes-related endothelial dysfunction in children with T1D [54,55,58], authors suggested that dysregulation of miRNA-21 in young patients with TD1 may serve as a marker of already existing renal fibrotic remodeling [45].

In fact, miRNA-126 is highly enriched in endothelial cells and platelets and can regulate VEGF-mediated effects, such as vascular integrity, angiogenesis, and wound repair [61]. It was demonstrated that miRNA-126 is expressed in glomerular and peritubular endothelial cells targeting Sprouty-related EVH1 domain containing protein (SPRED1) and phosphoinositol-3 kinase regulatory subunit 2 (PIK3R2), a negative VEGF pathway repressor [9]. In light of this, miRNA-126 has been considered as candidate marker of potential diabetes-related damage due to long-term high plasma glucose exposure [45,61].

Olivieri et al. [51] examined the deregulation of miRNA21-5p and 126-3p in 193 Italian patients with T2D and 107 healthy subjects, and further analyzed circulating levels of both miRNAs according to the presence of diabetic complications. Worthy of note, both miRNA levels dramatically decreased from controls to T2D patients without and with complications [51]. Interestingly, significantly greater levels of miRNA-21-5p and lower levels of miRNA-126-3p were demonstrated in patients with specific diabetic consequences such as myocardial infarction [45,51,59].

Importantly, research evidence has demonstrated that miRNA-126 was negatively linked to all diabetic consequences [62,63,64,65,66]. A cross-sectional nested case-control study [66] analyzed miRNA-126 expression in a cohort of 455 patients with T1D including 312 patients (as subjects with one or more complications of diabetes) and 143 control subjects (with no evidence of any complication). A significant correlation of miRNA-126 levels with each micro-/macrovascular diabetic complication was found, particularly for proliferative retinopathy [66].

Recently, a pathophysiological role for miRNA-377 has been reported in the onset of diabetes-related endothelial dysfunction [67,68]. Indeed, it may promote the expression of fibronectin in mesangial cells in hyperglycemic-induced oxidative stress [69].

In line with these data, El-Samahy et al. [46] compared urinary levels of mi-RNA216a and of miRNA-377 in 50 patients with T1D and 50 healthy controls. Patients with T1D were further divided into two groups according to urinary albumin-creatinine ratio as normoalbuminuric and with microalbuminuria. Both groups had significantly higher urinary miRNA-377 levels (*p* < 0.05) [46]. In particular, considerably higher concentrations were reported in the microalbuminuric group compared to normoalbuminuric patients (*p* = 0.001) [46]. Another elegant study on 70 children and adolescent with T1D demonstrated a significant increase in miRNA-377 levels in patients with DN [43]. Patients with DN also showed a significantly negative correlation between of miRNA-216a with creatinine (*p* = 0.049), while a positive correlation with eGFR when estimation was done using creatinine (*p* = 0.03) was described. This is in line with previous research supporting the occurrence of decreased urinary miRNA-216a levels in T1D patients with DN [46].

## 3. Evidence on the Role of mi-RNAs 25, -93, -210, -29, and -192 in DN Pathogenesis

Additional miRNAs have been found to contribute to DN development [42,43,62,68] (Table 2). In a recent study, the role of miRNA-25 has been investigated in a cohort of 70 pediatric patients with T1D that were divided into two groups: with or without DN (defined as increased or normal albumin creatinine ratio (ACR)) [43]. Authors found a statistically significant upregulation of miRNA-25 expression in the group without DN (*p* = 0.01), and its downregulation in the group with DN (*p* = 0.01) [43]. Moreover, miRNA-25 was reported to be negatively associated with ACR [43], suggesting a reno-protective for this miRNA through NADPH oxidase 4 (Nox4) inhibition [28,70]. Indeed, research has supported a pathogenic involvement of miRNA-25 in renal dysfunction by promoting oxidative stress [43,71].

Differently from miRNA-25, circulating plasma miRNA-93 levels were significantly up-regulated (*p* = 0.02) and positively correlated with ACR (*p* = 0.004) and HbA1c (*p* = 0.04) in patients with DN [43]. Interestingly, miRNA-93 was found to be a significant independent factor for the onset of albuminuria, suggesting its pathophysiological involvement in DN development [43]. In particular, studies revealed that overexpression of miRNA-93 might inhibit TGF-β1, induce endothelial to mesenchymal transition, and halt renal fibrogenesis via targeting ORAI 1 expression in human kidney 2 (HK2) cell lines [43,71,72]. Moreover, miRNA-93 inhibitors have been found to increase vascular endothelial growth factor (VEGF) secretion [72].

In the context of diabetes, miRNA-210 represents another circulating miRNA commonly known to be deregulated [45]. Indeed, it has been found to be upregulated under hypoxic and high glucose conditions in vitro and in patients with T2D, while its relationship with T1D still remains less defined [45,73].

In addition, other miRNAs have been involved in diabetic disease phenotypes. For example, miRNA-29 family (consisting of miRNA-29a/b/c sharing the same seed sequence) was largely investigated as a putative DN biomarker [28,45,70,71,72], since its protective role in fibrotic disease including kidney fibrosis [73]. Higher MiR-29 expression has been demonstrated in the main target tissues of insulin [74]. Both hyperglycemia and pro-inflammatory cytokines overexpressed miRNA-29 family which in turn repressed insulin-stimulated glucose uptake leading to insulin resistance [74]. In fact, its suppression with anti-miRNA-29 oligomers has been found to be protective against DN [73], further supporting miRNA-29 as biomarker for DN and atherosclerosis in T2D [73]. In agreement with the study by Wang et al. [75], it has been demonstrated that TGF-β1 reduced the expression of the miR-29 family in proximal tubular cells in a hyperglycemic context resulting in the classic epithelium-like to mesenchymal transition (EMT)-like morphologic changes, including significantly increased expression of collagens I and IV [28]. An elegant study by Chen et al. [12] concluded that miRNA-29b may have a protective effect in diabetic kidney disease in db/db mice by inhibiting TGF-β/small mother against decapentaplegic (SMAD) 3 signaling pathway and specific protein 1/NF-κB-driven renal inflammation [28]. Additional research corroborated the proposal of miRNA-29c as novel target in DN [76] since its antifibrotic role inducing cell apoptosis and extracellular matrix protein accumulation via TGF-β signaling pathway [76]. Taking into account the critical contribution of endothelial-to-mesenchymal transition (EndMT) to the protective role of miRNAs in DN development, interventions preventing this crosstalk are crucial for the treatment of the disease [77,78]. For instance, Dipeptidyl Peptidase 4 (DPP-4) inhibitors have been proposed as potential therapeutic options [77]. Indeed, their potential pathogenic role might be attributable to the suppression of the EndMT-driven TGF-β signaling in diabetic kidneys and the subsequent upregulation of miRNA-29 family expression [77].

Worthy of note, additional miRNAs pathogenically linked to DN have been suggested as novel promising early biomarkers. Evidence on animal models and adult subjects with T2D demonstrated an inverse correlation of miRNA 146a expression with glomerular damage, suggesting a potential protective role for podocytic miR-146a in DN development [79,80]. Similarly, circulating miRNA-130b levels were found to be reduced in patients with a more pronounced decrease in the subgroup with microalbuminuria [81], Besides, upregulation of miRNA-424 expression was found to reduce pathological renal changes (e.g., cellular apoptosis) occurring in DN [82].

In this tangled landscape, the role of miRNA-192 in DN progression needs to be also considered [83,84,85]. miRNA-192 represents one of the most abundant miRNAs in the kidney implicated in the development of matrix accumulation by controlling TGF-β-induced collagen type 1 α-2 (COL1A1 and -2) expression through the downregulation of E-box repressors zinc finger E-box-binding homeobox (ZEB)1 and 2 [86]. Evidence reported that miRNA-192 expression was significantly lower in patients with T2D compared to healthy controls and in patients with microalbuminuria compared to those with normoalbuminuria [84]. A recent pediatric work examined the role of miRNA-192 and its relationship with Serum Klotho [KL] as potential target for DN treatment [47]. Alpha-Klotho is a co-receptor for fibroblast growth factor (FGF)-23 involved in the regulation of oxidative stress, inflammation, and fibrosis by inhibiting insulin/insulin-like growth factor-1 (IGF-1) and TGF-β1 signaling pathways [87,88]. In the examined cohort, KL was significantly associated with HbA1c at time of evaluation (*p* = 0.037), HbA1c mean over 2 years (*p* = 0.007), and diabetes duration (*p* < 0.001) [47]. Serum miRNA-192 concentrations were negatively associated with circulating KL levels in children with prolonged duration of diabetes, suggesting a potential regulatory role for miRNA-192 in soluble KL expression [47]. Additionally, given its role in modulating certain pathophysiological processes including oxidative stress, senescence, and inflammation, in vitro evidence also supported a key role for miRNA-192 in diabetic complications [47,89].

## 4. Conclusions and Future Perspectives

Unravelling the underlying pathophysiological mechanisms of DN is essential to improve the overall management of the disease through the identification of novel biomarkers and insightful therapeutic targets. Although there is substantial evidence on the contribution of certain miRNAs in DN development and progression, more scientific efforts including validation with large-cohort prospective studies in future are needed to overcome the challenge of a deeper understanding of their potential diagnostic, therapeutic, and prognostic role, and of the translation of their therapeutic potential into clinical application. On this basis, the large availability of adult evidence in the field might offer guidance for meaningful pediatric studies. In light of the serious burden of DN since childhood, current pediatric knowledge in the intricate research area of early diagnosis and targeted therapy of DN needs to be expanded.

As a future insightful perspective, a whole miRNAs signature of DN in childhood might pave the way for significant clinical improvements in the overall management of these at-risk patients.

## Figures and Tables

**Table 1:** Main findings of the studies on the role of miRNAs377, -126, and -21 in DN.

miRNA	References	Study Design	Population	Main Findings
miRNA 377	[43]	Cross-sectional study	70 children and adolescent with T1D from Diabetes, Endocrine and Metabolism Pediatric Unit (DEMPU) Children Hospital, Cairo University. Mean age 13.21 ± 3.66 years. -25 without DN -45 with DN	Serum miRNA377 was significantly higher in patients with DN than in those without DN and it was positively correlated with LDL cholesterol.
	[46]	Cross-sectional study	50 patients with T1D compared to 50 healthy controls from Pediatric Diabetes Clinic, of Pediatric Hospital, Ain Shams University. Mean age 13.7 ± 3.3 years. T1D patients were divided in two groups: -group A: 26 normoalbuminuric diabetic patients without nephropathy. -group B: 24 diabetic patients with nephropathy.	Compared to healthy controls, both patients with and without DN had significantly higher urinary miRNA-377 levels (*p* ≤ 0.05). In the analyzed diabetic cohort, urinary miR-377 expression was considerably higher in the group B (*p* ≤ 0.001).
miRNA-126	[48]	Cross-sectional nested case-control study	455 patients with T1D. Case subjects (*n* = 312): patients with ≥1 complications of diabetes; control subjects (*n* = 143): individuals with no evidence of any complication.	miRNA-126 was negatively linked to each micro-/macrovascular complication that was independently analyzed and with all complications (OR = 0.85, 95% CI 0.75–0.96).
	[45]	Cross-sectional observational cohort study	-68 young patients with T1D (age 6–18 years) and 79 age- and gender-matched healthy subjects.	Patients with T1D had considerably lower urinary miRNA-126 levels than age- and gender-matched controls. There was a negative correlation between HbA1c mean and miRNA-126 levels.
miRNA-21	[45]	Cross-sectional observational cohort study	68 patients with T1D Age 6–18 years, duration of disease >1 year, C-peptide < 0.3 nmol/L, and intensive insulin treatment with either multiple daily insulin injections or continuous subcutaneous insulin infusion for at least 6 months.	A significant increase in miRNA-21 in plasma (*p* = 0.008) and urine (*p* ≤ 0.0001) of patients with T1D was found. A positive correlation between urinary miRNA-21 and CRP (r = 0.298, *p* = 0.029) was showed.
	[43]	Cross-sectional study	70 children and adolescents with T1D.	miRNA-21 was positively correlated with urinary levels of cystatin c (r = 0.6, *p* = 0.01) and negatively correlated with eGFR using cystatin c (r = −0.6, *p* = 0.01).

Abbreviations: T1D: type 1 diabetes; T2D: type 2 diabetes; miRNA: microRNA; DN: Diabetic Nephropathy; ACR: albumin creatinine ratio; HbA1c: Glycosylated hemoglobin; CRP: C-reactive protein; eGFR: Estimated Glomerular Filtration Rate.

**Table 2:** Main findings of the studies on the role of miRNAs 25, -93, -210, and -126 in DN.

miRNA	Reference	Study Design	Population	Main Findings
miRNA-25	[43]	Hospital-based cohort cross-sectional study	70 patients with T1D with a 5 years’ duration of diabetes or more -25 patients (16 males and 9 females) without DN (normal ACR) -45 patients (19 males and 26 females) with DN (increased ACR).	miRNA-25 may have a reno-protective role. Negative correlation between miRNA-25 and ACR was found. miRNAs-25 levels were upregulated in the group without DN and downregulated in the group with DN.
miRNA-93	[43]	Hospital-based cohort	70 T1D patients with a 5 years’ duration of diabetes or more -25 patients (16 males and 9 females) without DN (normal ACR) -45 patients (19 males and 26 females) with DN (increased ACR).	A positive correlation between miR-93 and HbA1c and ACR was reported. Up-regulation of miRNA-93 in the group with DN compared to the group without DN was found.
miRNA-210	[45]	Cross-sectional observational cohort study	68 patients with T1D Age 6–18 years, duration of disease > 1 year, C-peptide < 0.3 nmol/L, and intensive insulin treatment with either multiple daily insulin injections or continuous subcutaneous insulin infusion for at least 6 months.	Both plasma and urinary miRNA-210 levels of patients with T1D were higher than controls.
miRNA-126	[45]	Cross-sectional observational cohort study	68 patients with T1D Age 6–18 years, duration of disease > 1 year, C-peptide < 0.3 nmol/L, and intensive insulin treatment with either multiple daily insulin injections or continuous subcutaneous insulin infusion for at least 6 months.	No differences emerged in plasmatic T1D sample, while lower miRNA-126 levels were confirmed in urine T1D samples compared to controls (*p* = 0.016). A negative association between urinary miRNA-126 levels and HbA1c was found.

Abbreviations: T1D: type 1 diabetes; T2D: type 2 diabetes; miRNA: microRNA; DN: Diabetic Nephropathy; ACR: albumin creatinine ratio; HbA1c: Glycosylated hemoglobin; CRP: C-reactive protein; eGFR: Estimated Glomerular Filtration Rate.

## Data Availability

Not applicable.
